# Dietary patterns associated with colorectal cancer risk in the Malaysian population: a case–control study with exploratory factor and regression analysis

**DOI:** 10.1186/s12889-023-16283-6

**Published:** 2023-07-19

**Authors:** Ainaa Almardhiyah Abd Rashid, Lydiatul Shima Ashari, Nor Hamizah Shafiee, Raja Affendi Raja Ali, Lee Yeong Yeh, Mohd Razif Shahril, Hamid Jan Jan Mohamed

**Affiliations:** 1 Nutrition Programme, School of Health Sciences, Health Campus, Universiti Sains Malaysia, Kubang Kerian, Kelantan 16150 Malaysia; 2 Department of Medicine, Faculty of Medicine, Universiti Kebangsaan Malaysia, Jalan Yaacob Latif, Bandar Tun Razak, Kuala Lumpur, Cheras 56000 Malaysia; 3 Gut Research Group, Faculty of Medicine, Universiti Kebangsaan Malaysia, Jalan Yaacob Latif, Bandar Tun Razak, Kuala Lumpur, Cheras 56000 Malaysia; 4 School of Medical and Life Sciences, Sunway University, Jalan Universiti, Bandar Sunway, Selangor 47500 Malaysia; 5 Department of Medicine, School of Medical Sciences, Universiti Sains Malaysia, Health Campus, Jalan Raja Perempuan Zainab 2, Kubang Kerian, Kelantan 16150 Malaysia; 6 GI Function & Motility Unit, Hospital USM, Jalan Raja Perempuan Zainab 2, Kubang Kerian, Kelantan 16150 Malaysia; 7 Centre for Healthy Ageing and Wellness (HCARE), Faculty of Health Sciences, Universiti Kebangsaan Malaysia, Jalan Raja Muda Abdul Aziz, Kuala Lumpur, 50300 Malaysia

**Keywords:** Colorectal cancer, Dietary patterns, Processed diet, Western diet, Prudent diet

## Abstract

**Background:**

Studies on the relationship between diet and colorectal cancer (CRC) risk using single food or nutrient approach are widely conducted as opposed to dietary pattern approach. Therefore, this study aimed to determine the major dietary patterns and their association with CRC risk among Malaysians.

**Methods:**

Patients aged between 18 and 80 years old from two teaching hospitals in Peninsular Malaysia were recruited through purposive sampling. Socio-demographic information and anthropometry data were assessed before the colonoscopy procedure, and dietary intake was also recorded using a validated semi-quantitative food frequency questionnaire (FFQ). Cases were those patients having histopathologically proven CRC, while controls were those without.

**Results:**

Four major dietary patterns were identified: the allergenic diet, plant-based diet, processed diet, and energy-dense diet pattern. After adjusting for potential covariates, the processed diet pattern was consistently associated with CRC (OR = 3.45; 95% CI = 1.25–9.52; *P* = 0.017) while the plant-based diet, energy-dense diet, and allergenic diet were not associated with CRC risk.

**Conclusions:**

The processed diet pattern attributed to a diet high in confectionaries and fast foods was associated with an increased risk of CRC in the Malaysian population. In order to give prevention measures through lifestyle change, more research could be done on the effect of food patterns on faecal microbiota associated with CRC.

## Background

Colorectal cancer (CRC) has a five-year prevalence rate of about 5.3 million people worldwide. With estimates of over 1.9 million and 935,000 fatalities, respectively, CRC is the second most prevalent cause of cancer death and the fourth most common malignancy in terms of diagnoses [1]. A study in European countries showed that the incidence of CRC in Austria, Germany, and the Czech Republic reduced over time [2]. For Denmark, Slovenia, and the Netherlands, the age-standardised incidence rates primarily showed an increment but then subsequently reduced, while the rate is increased for Bulgaria, Norway, Estonia, and Ukraine countries. The age-standardised incidence rates of CRC, on the other hand, were constant and barely varied from 17.03 to 20.01 per 100,000 in northern Malaysia, whereas the age-standardized mortality rates dropped from 12.73 per 100,000 in 2008 to 2.99 per 100,000 in 2017 [3].

There are three main risk factors for CRC, including family and personal medical history, lifestyle and others [4]. Family and personal medical history included family history and genetics, inflammatory bowel disease, colon polyps, diabetes mellitus, and cholecystectomy [4]. As for lifestyle factors, some of these included dietary patterns, overweight and obesity, physical inactivity, smoking, and alcohol intake, while others address gut microbiota, age, gender, race, and socioeconomic status. [4]. Dietary intake has become one of the important factors contributing to CRC development. Populations consuming high intakes of red meat [5], sugar [6], and white bread [7] may be at increased risk of the disease.

Several studies had been conducted to assess the relationship between diet and CRC risk [5, 7, 8]; however, those studies used a single food or nutrient approach rather than a dietary pattern approach. Dietary pattern is more preferred because it involves quantification, variation, or the combination of different foods and beverages in the diet and their habitual consumption frequency [9]. In addition, dietary pattern analysis also is a crucial new technique that may help to explain the relationship [10]. The application of this dietary pattern to non-western population is limited, due to lack of studies in Asian populations evaluating the association between dietary pattern and CRC risk [6, 11–15]. To the best of our knowledge, there is no study on dietary patterns for the determination of CRC risk in Malaysia. Therefore, the aim of this study was to determine the major dietary patterns and their association with CRC risk among the Malaysian population.

## Methods

### Study design and study population

This hospital-based case–control study was conducted between July 2020 and January 2022 in two teaching hospitals, namely Hospital Universiti Sains Malaysia (HUSM) and Hospital Canselor Tuanku Muhriz Universiti Kebangsaan Malaysia (HCTM UKM). HUSM is located in Kota Bharu (northeastern region of Peninsular Malaysia) with a population density of 122 people per square kilometer, while HCTM UKM is located in Kuala Lumpur (Klang Valley of Peninsular Malaysia) with an 8,045-population density per square kilometer. These tertiary teaching hospitals represented urban (HCTM UKM) and suburban (HUSM) areas, respectively [16]. Around 126 patients (30 cases and 96 controls) were enrolled from HUSM and 138 patients (49 cases and 89 controls) from HCTM UKM.

### Sample size

The sample size in this study was computed using Power and Sample software (PS software) version 3.0 by William D. Dupont and Walton D. Plummer (1997–2009). The study from Safari et al*.* (2013) was adopted as it has the most similar criteria to this study [12]. A total of 78 cases and 156 controls (plus an additional 30% of non-respondents) were needed for the investigation, assuming the expected proportions in case and control were 0.606 and 0.387, 80% study power, alpha 0.05, and a participation ratio of 1:2.

### Patient selection

The study protocol was reviewed and approved by the Human Research Ethics Committee of Universiti Sains Malaysia (USM/JEPeM/19060354) and the Universiti Kebangsaan Malaysia Medical and Research Ethics Committee (UKMREC; FF-2020–005). All patients who attended the colonoscopy procedure were approached in a sequential manner, and informed consent was obtained from those who agreed to participate. Inclusion criteria were Malaysian, not being on any special diet that could influence their weight status, have no previous abdominal surgeries associated with bowel obstruction, and females must not be pregnant or breastfeeding. Aged 18 to 80 were selected as the cancer incidence increased after the age of 30 years while the CRC incidence increased after the age of 60 years [17].

After completing the colonoscopy procedure and obtaining the results of histopathology, patients were assigned to the case and control groups. Thus, cases were endoscopically and histopathologically confirmed CRCs, diagnosed no more than one year before study enrolment, and had no previous cancer diagnosis at other anatomic sites. They also did not have severe mental disorders, including major depression, schizophrenia, and anxiety, or physical disabilities affecting independent self-care and diet. Meanwhile, controls were patients with histological tubular adenoma and normal or benign findings such as diverticulum, colitis, appendicitis, and a rectal ulcer.

### Data collection

Patients would complete a face-to-face interview in the Malay language during their hospital visit using the same structured questionnaire at both study sites. It consisted of socio-demographics data (age, gender, ethnicity, marital status), smoking status, supplement intake, employment status, household income, educational level, medical and family history of CRC, anthropometric parameters, and dietary intake.

### Anthropometric measurements

Anthropometric measurements were taken in accordance with standard procedure [18]. During the measuring process, two researchers were incorporated as measurers and recorders. Only one measurer was involved in the measuring process to minimize inter-measurer bias. The average values were finalized from the duplication measurements taken.

Height was measured using a stadiometer (Seca, 217, Hamburg, Germany) with the respondent standing straight and barefoot, and the reading was measured to the nearest 0.5 cm. Then, weight and body composition were measured using the body composition analyzer [19] which produced estimated values of the measurements taken using the dual-energy X-ray absorptiometry method (DEXA) and bioelectrical impedance analysis (BIA). The obtained height and weight were used to calculate BMI by using the formula: BMI = weight (kg)/height (m^2^). The BMI cut-off point was classified according to the criteria defined by the World Health Organization (WHO) [20].

A measuring tape was used to measure the waist circumference (WC) and hip circumference (HC) (Seca, 201, Hamburg, Germany). Respondent stood straight with arms hanging by the sides. The tape was positioned at the midpoint between the inferior margin of the last rib and the iliac crest, and then passed firmly around the waist, not compressing the skin. The measurement for WC was taken at the end of expiration when the respondent breathed normally, to the nearest 0.1 cm. HC was measured by adjusting the tape level to the adjudged level of the greatest posterior protuberance of the buttocks. The measurement was taken to the nearest 0.1 cm after it was perpendicular to the trunk's long axis.

### Dietary Intake Questionnaire Via FFQ

The validated semi-quantitative food frequency questionnaire (FFQ) consisted of 142 food items from 16 food groups. It is basically a modification of food list related to CRC from the original National Health and Morbidity Survey (NHMS) 2014 questionnaire [21]. The validity and reproducibility were tested, and the FFQ was good for estimating absolute nutrient and food group intakes. The 16 food groups included cereal products, meats, fish, and seafood, eggs, vegetables, legumes, bread spreads, fruits, confectionaries, fast foods, non-sugary drinks, sugar-sweetened drinks, alcoholic drinks, condiments, and dairy products.

The diet assessed was for a period of one year prior to the colonoscopy procedure, which will capture the dietary pattern of cases before cancer diagnosis and the habitual dietary pattern of the controls. During data collection, patients were required to report the frequency of foods and drinks consumed and the amount intake on a daily, weekly, monthly, and yearly/never basis through a face-to-face interview. Photographs of household measurements, including glass, cup, tablespoon, teaspoon, and scoop were provided to aid respondents in estimating the portion sizes of the foods that they consumed.

To obtain the energy and nutrient values, the daily intake of each food item was calculated using the frequency of intake per day x total number of servings x weight of food in one serving. All the dietary information from the semi-quantitative FFQ was analyzed using Nutritionist Pro™ Diet Analysis Software, version 7.8.0 (Axxya Systems, version 2020, Redmond, USA), and the database selected was Nutrient Composition of Malaysian Foods. Recipes that were not available in the reference list were added to the database, where the portion sizes were calculated based on standard recipe sizes (total serving and per serving size). The Atlas of Food Exchanges and Portion Sizes, the Nutrient Composition of Malaysian Foods, the Malaysian Food Album [22], and the Malaysian Food Composition Database (MyFCD) [23] were used to determine the weight of foods or ingredients used in the recipes. The nutritional content of the food product was obtained from its packaging or MyFCD and was inserted into the database.

However, under-reported and over-reported dietary intake were eliminated using the Goldberg cut-off based on confidence limit (CL) of physical activity level (PAL) and the ratio of energy intake (EI) and basal metabolic rate (BMR) [24].

### Exploratory factor analysis

All data analysis was performed using IBM SPSS Statistics, Version 26.0 (Chicago, IL, USA), and statistics were considered significant with a *p*-value < 0.05. Firstly, based on the functions of food and its nutritional properties, the 142 food items in the FFQ would be classified into the predetermined 16 food groups. Then, using exploratory factor analysis (EFA), the identified dietary factors were rotated using an orthogonal varimax rotation approach for the maximum amount of variance explained and the uncorrelated components for improved interpretability. The following criteria were used to determine which factors should be kept: factor interpretability, factor eigenvalues larger than 1.15, scree plot break point, and proportion of variance explained [25]. Food groups with absolute rotation factor loadings equal to or higher than 0.30 were used to designate dietary patterns. This highlights the significance of factor loading when calculating factor scores that correspond to each pattern individually. Furthermore, each patient will receive a factor score determined by a loading matrix for each dietary pattern (factor) by indicating how well their diet matched that pattern. In other words, a person with a higher factor score adhered to that pattern more strongly.

### Statistical analyses

To determine the association between dietary pattern and CRC, unconditional logistic models were initially used, and only age and total energy consumption were accounted for in Model 1. Scores of dietary pattern were divided into tertiles with the lowest tertiles serving as the reference group [26]. Additional confounding factors were adjusted using the multiple logistic regression analyses, including age, total daily calorie intake, gender, BMI, marital status, level of education, and history of CRC in the family (referred to as Model 2). These factors were chosen based on the findings of the systematic review and meta-analysis [27]. Risks were reported as odds ratios (OR) and 95 percent confidence intervals (CI). All data analysis was performed using IBM SPSS Statistics, Version 26.0 (Chicago, IL, USA), and statistics were considered significant with a *p*-value < 0.05.

## Results

### Recruitment and dietary assessment finalization

**Fig. 1:**
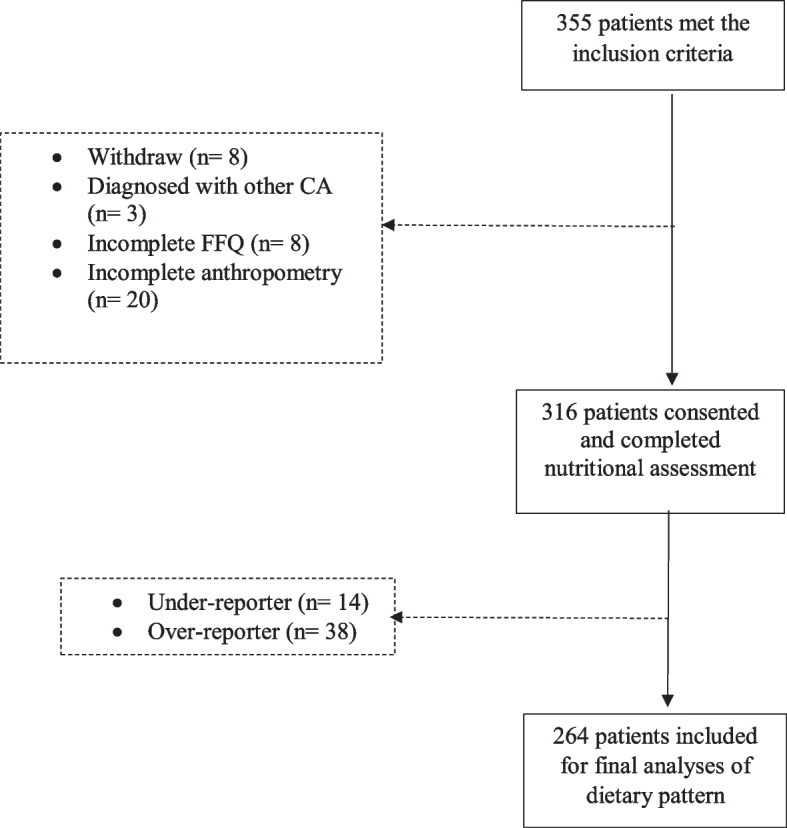
Flow chart indicating screening, recruitment, and dietary assessment finalization. CA, cancer; FFQ, food frequency questionnaire

Initially, 355 eligible patients were recruited, but 28 did not complete the nutritional assessment (*n* = 20 for anthropometry and *n* = 8 for FFQ), 8 withdrew from the study, and 3 were diagnosed with other cancers (brain, urinary bladder, and small intestine). Out of the remaining 316 patients, 52 did not report a plausible intake which consisted of 14 patients underreported and 38 patients overreported their dietary intake. Hence, the final total of patients was 264 (79 cases and 185 controls) (Fig. 1).

### Baseline characteristics of patients

Patient baseline characteristics are shown in Table 1. Cases were significantly older than controls (61.0 years ± 11.8 vs. 50.8 years ± 15.4) (*p* < 0.001) and were mostly Malay as compared to controls of Chinese and Indian ethnicity.

**Table 1:** Baseline characteristics of the study patients (*n* = 264)

Variables	Cases, n (%)	Controls, n (%)	*p*-value
Age (years)^a^	61.0 (11.8)	50.8 (15.4)	** < 0.001**
**Gender**			0.955
Male	43 (54.4)	100 (54.1)	
Female	36 (45.6)	85 (45.9)	
**Ethnicity**			**0.003**
Malay	63 (79.7)	122 (65.9)	
Chinese	16 (20.3)	40 (21.6)	
Indian	0 (0)	23 (12.4)	
**Study site**			**0.042**
Urban area	49 (62.0)	89 (48.4)	
Suburban area	30 (38.0)	96 (51.6)	
**Marital status**			**0.016**
Single	7 (8.9)	37 (20.0)	
Married	49 (62.0)	117 (63.2)	
Divorced/ widowed	23 (29.1)	31 (16.8)	
**Education**			0.116
Primary	13 (16.5)	20 (10.8)	
Secondary/ college	39 (49.4)	73 (39.5)	
Higher institution	27 (34.2)	92 (49.7)	
**Family history of CRC**^b^			**0.018**
First degree relative	23 (29.5)	30 (16.5)	
Second degree relative	10 (12.8)	15 (8.2)	
Distant relative	3 (3.8)	3 (1.6)	
No family history CRC	42 (53.8)	134 (73.6)	
**Occupation**			0.074
Government/ semi-government worker	20 (32.7)	66 (35.9)	
Private sector worker	11 (13.9)	35 (19.0)	
Self-employed/ Own business	8 (10.1)	24 (13.0)	
Housewife	14 (17.7)	16 (8.7)	
Unemployed	4 (5.1)	11 (6.0)	
Pensioner	22 (27.8)	32 (17.4)	
**Monthly household income (MYR)**^**c**^			0.128
< 3860	50 (63.3)	99 (53.8)	
3861–8319	27 (34.2)	69 (37.5)	
≥ 8320	2 (2.5)	16 (8.7)	
**Smoking status**			0.606
Active smoker	15 (19.0)	34 (20.5)	
Former smoker	17 (21.5)	44 (26.5)	
Nonsmoker	47 (59.5)	88 (53.0)	
**Vitamin/ mineral supplement intake**			** ≤ 0.001**
Yes	46 (59.0)	42 (23.1)	
No	32 (41.0)	140 (76.9)	
**BMI**			**0.004**
Underweight	49 (62.0)	78 (42.2)	
Normal	4 (5.1)	5 (2.7)	
Overweight/Obese	26 (32.9)	102 (55.1)	
WC (cm), male^a^	89.4 (16.1)	97.1 (13.1)	**0.004**
WC (cm), female^a^	85.4 (13.8)	90.2 (15.6)	0.115
HC (cm), male^a^	96.8 (5.4)	101.7 (7)	** ≤ 0.001**
HC (cm), female^a^	101.3 (13.6)	103.9 (10.7)	0.267
WHR (cm), male^a^	0.9 (0.2)	1.0 (0.1)	0.245
WHR (cm), female^a^	0.8 (0.1)	0.9 (0.1)	0.182
Body fat %, male^a^	24.5 (6.7)	26.7 (6.3)	0.069
Body fat %, female^a^	31.8 (7.5)	34.5 (8.4)	0.096
Energy intake (kcal)^a^	1881.4 (396.9)	1946.0 (362.3)	0.199

*p*-value was calculated using the Chi-Square test

^a^Data presented in mean (SD) and p-value was calculated by the independent T-test

^b^Sample size was not always *n* = 264 due to missing values

^c^Based on the cut-off of Eleventh Malaysia Plan (2015)

In addition, vs. controls, cases were more likely to be from an urban area (*p* = 0.042), divorced or widowed (*p* = 0.016) and have a positive family history of CRC from the first-, second-, and distant relatives (*p* = 0.018). The proportions of BMI categories (*p* = 0.004), WC and HC in males (*p* = 0.004 and *p* ≤ 0.001, respectively) were significantly different between cases and controls. Case group significantly had lean BMI (underweight 62.0% and normal weight 5.1%) and lower WC (89.4 cm ± 16.1) and HC (96.8 cm ± 5.4) among males as compared to control group.

### Identification of dietary patterns based on EFA

**Fig. 2:**
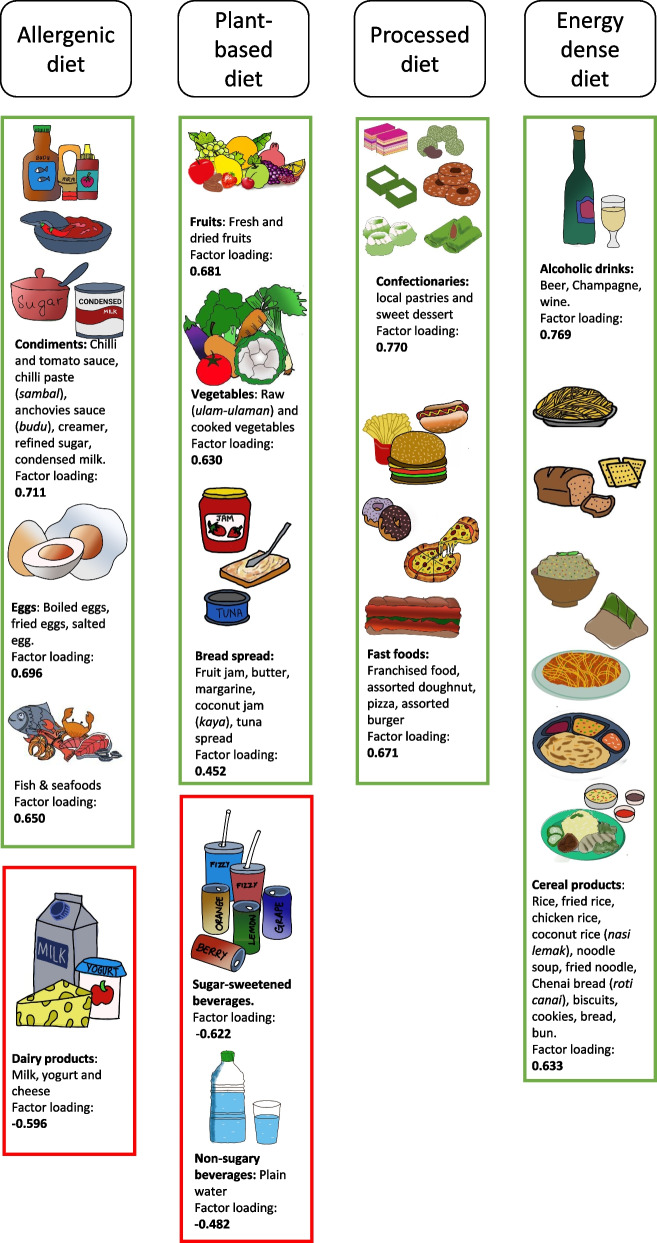
Food groups within four dietary patterns with positive and negative loadings. Absolute values less than 0.2 were not listed for simplicity, and those above 0.3 were presented to visually emphasize the strength of association. The green box indicates positive factor loading, and the red box indicates negative factor loading

Four major dietary patterns were identified from EFA and factor labeling as shown in Fig. 2. A predefined food group was considered loaded on a specific pattern when its absolute factor loading was ≥ 0.3. The first was the allergenic diet pattern, characterized by high loadings for condiments, eggs, fish and seafood, and dairy products. The second was the plant-based diet pattern, loaded heavily on fresh fruits, dried fruits, raw vegetables, cooked vegetables, and bread spread. Next, the third was the processed diet pattern, which comprised of confectionaries and fast foods. The fourth was an energy-dense diet pattern with high loadings of alcoholic drinks and cereals.

### Association of dietary patterns with CRC

Table 2 reveals the odds ratios and their 95% confidence intervals for CRC by the tertiles of factor scores. After adjusting for potential covariates, the processed diet pattern was found to be associated with CRC (OR = 3.45; 95% CI = 1.25–9.52; *P* = 0.017). However, when adjusting with only age and total energy intake as confounders, the allergenic diet pattern and the processed diet pattern were found to be associated with CRC (OR = 2.34; 95% CI = 1.12–4.91; *P* = 0.024; OR = 2.68; 95% CI = 1.06–6.77; *P* = 0.037). The plant-based diet and energy-dense diets were not significantly associated with CRC (p > 0.05).

**Table 2:** Odds ratios and 95% CI of colorectal cancer according to the four major dietary patterns

**Dietary pattern**	Tertiles				
	T1	T2		T3	
		OR	95% CI	OR	95% CI
**Allergenic diet**					
^d^Model 1	1.00	1.12	0.52, 2.41	**2.34**	1.12, 4.91
^e^Model 2	1.00	0.64	0.27, 1.52	1.34	0.61, 3.14
**Plant-based diet**					
^d^Model 1	1.00	1.60	0.83, 3.09	0.68	0.32, 1.46
^e^Model 2	1.00	1.43	0.69, 2.97	0.45	0.19, 1.04
**Processed-diet**					
^d^Model 1	1.00	1.43	0.68, 2.99	**2.68**	1.06, 6.77
^e^Model 2	1.00	1.27	0.57, 2.84	**3.45**	1.25, 9.52
**Energy-dense diet**					
^d^Model 1	1.00	0.68	0.33, 1.38	0.45	0.19, 1.05
^e^Model 2	1.00	0.54	0.25, 1.15	0.53	0.21, 1.31

^d^Adjusted for age and total energy intake, ^e^Adjusted for: age, total energy intake, gender, body mass index, marital status, education, and family history of CRC; significant associations are in bold

## Discussion

The current findings provided the first evidence of a link between higher factor loading, which reflected dietary patterns with greater potential food groups, and an elevated risk of CRC in a multi-ethnic Malaysian population. The findings of this population, whose dietary choices differ markedly from those of the more well-studied populations in Western countries, reaffirm that the use of the EFA as a tool for relating the dietary pattern to CRC in a wide range of populations. This study’s findings added to the corroboration that some dietary patterns are incorporated with the CRC studies in United States [25, 28–35], European [36–39], and Asian populations [14, 40–43]. Thus, four dietary patterns were identified from 16 food groups, including the allergenic diet, plant-based diet, processed diet, and energy dense diet. Then, the processed diet was found to be associated with CRC in the Malaysian population.

Predominantly, previous reported studies associated with CRCs have classified the dietary pattern into two groups: the western/unhealthy pattern and the prudent/healthy pattern [29, 30, 35, 44–48]. This current study found that processed diet being associated with CRCs would be considered the western/unhealthy pattern, and the food groups loaded in both patterns shared similarities [38]. The processed diet pattern included confectionaries and fast foods, may have contributed to the patterns known as light meals, Japanese meals, animal food patterns, and high-dairy, high-fruit and vegetable, high-starch and low-alcohol diets (DFSA) [27]. A multicase-control study from 11 Spanish provinces had primarily several studies to assess associations of dietary pattern with gastric, breast and prostate cancer. Another study was reconstructed to observe the association between dietary patterns and CRC. Results produced were consistent with the primary previous study which showed that the western dietary pattern had putative risk for breast and gastric cancer [49]. In addition, western dietary patterns are associated with an increased risk of CRC, particularly distal colon and rectal tumors [35].

It is evident from this study that the processed diet consisted primarily of fast food (particularly processed meat and franchised meals) and confectionaries (mainly refined carbohydrates and sugars from local pastries and sweet desserts). A population-based case–control study in Ontario, Canada, found that westernized dietary patterns were associated with a statistically significant increased risk of early onset CRC. Their western-like dietary pattern of derivation consists of red meat, processed meat, sugary drinks, sugary desserts, fast food, and processed snacks [50]. Meanwhile, higher consumption of ultra-processed foods (meat, poultry, and seafood-based ready-to-eat products) among men and ready-to-eat/heat mixed dishes among women was associated with increased risk of colorectal cancer in three prospective US cohort studies [51]. A systematic review proved that a raised of CRC development from six cohort studies with adherence to western dietary pattern which comprised of refined grain and red and processed meat [52].

A meta-analysis with twenty-eight studies reported an increase in CRC risk (relative risk (RR) = 1.25; 95% CI = 1.11–1.40; *P* < 0.001) when compared to the western vs. prudent dietary patterns. This confirmed that a diet rich in meats (red and processed), refined grains and sugar-rich food is linked with an increased risk of CRC [27]. This result was also supported by the previous review that found the harms of a diet high in meats, refined grains, and added sugar [53]. Moreover, a recent systematic review of meta-analyses on whole grain and refined grain concluded refined grains was related to gastric and colon cancer [54]. However, most studies covered in that meta-analyses had no exact definition of refined grains. This led to poor outcomes because refined grains were commonly referred to both staple refined grain and indulgent refined grain foods [54]. Bread, cereal, pasta, and rice are examples of staple refined grains, as are cakes, cookies, pastries, sweets or grain-based desserts which are indulgent refined grains [55]. It is necessary to interpret results from the perspective of how refined grains have been defined [55].

A case–control study conducted in Iran showed that sweets and desserts (including pastry), combined with red meat, soft drinks, and high-fat dairies, had higher factor loading in the western dietary pattern and was associated with CRC [56]. This is in line with the current study that showed fast foods, local pastries, and sweet desserts were associated with CRC. An Iranian pattern was significantly associated with an increased odds of CRC which derived from processed meat (sausages, hamburger and salami) and sweets and dessert (chocolate, dry sweet, cookies, cakes, pastry, jam, honey, halvah) [57]. In addition, an Iran case–control study found significant associations were reported between different types of dietary carbohydrates: maltose (OR = 9.03, CI 95%: 3.93–20.78, *P* < 0.001), fructose (OR = 1.31, CI 95%: 1.19–1.43, *P* < 0.001), galactose (OR = 1.31, CI 95%: 1.07–1.6, *P* = 0.008), sucrose (OR = 1.19, CI 95%: 1.12. − 1.25, *P* < 0.001), glucose (OR = 1.06, CI 95%: 1.01–1.11, *P* = 0.009), sugar (OR = 1.02, CI 95%: 1.01–1.03, *P* < 0.001), lactose (OR = 1.009, CI 95%: 1.01–1.18, *P* = 0.02) and carbohydrate (OR = 1.009, CI 95%: 1.003–1.01, *P* = 0.002) [58]. However, a prospective Japanese cohort study (Asian population) among middle-aged adults showed insignificant association of all sugar types with CRC risk except a positive association of total sugar intake in women with rectal cancer specifically [59].

Mechanisms of these diets in the causation of CRCs have been studied widely. Various molecules are involved in the causation, including heme iron, N-nitroso-compound (NOC), heterocyclic amines (HCAs), and polycyclic aromatic hydrocarbons (PAHs) [60]. Various theories have also been determined on the potential carcinogenicity of red and processed meat. The probable involvement of PAHs and HACs in the carcinogenesis brought on by DNA mutation is described via a proposed mechanism. Another theory, heme produces cytotoxic and genotoxic aldehydes that leads to the development of cancer by causing lipid peroxidation and the subsequent creation of NOCs [61]. Sugar consumption was linked to inflammation or angiogenesis, as demonstrated in the recent global ColoCare Study [62]. In addition, raised plasma insulin levels and insulin-like growth factor-1, both known to promote tumor growth [27, 63].

This study has several strengths. First, this study represents the Malaysian population with different socio-economic background because the recruitment was conducted from two different geographical location. Each site was west and east Peninsular Malaysia and at the same time, covering urban and suburban regions. Second, the dietary questionnaire used in the current study was specifically modified and validated for this study [21]. Third, patients with dietary reporting bias have been omitted using the Goldberg equation. Fourth, instead of a single nutrient or food approach, EFA was used to derive new and non-correlated variables in order to explain the variations in dietary habits. This allowed the researcher to obtain a more comprehensive and accurate picture of dietary exposures in a population. Finally, when exploring the relationships between dietary patterns and CRC, a multivariate logistic regression model that controlled for a wide range of potential confounding factors was fitted.

There are several noteworthy limitations. First, the purposive sampling can be prone to difficulty in defending the population representativeness, but this has been lessened by recruiting patients from different study sites. Second dietary patterns we observed may not be comparable with other studies due to variances in dietary patterns based on ethnicity, culture, religion, geography, and other socioeconomic determinants. Third, one year of dietary recall might cause memory bias, and overreporting is common when using the FFQ. Thus, this limitation has been minimized by excluding implausible dietary reporters.

## Conclusion

The present study has identified four dietary patterns from 16 food groups in the Malaysian diet, including an allergenic diet, a plant-based diet, a processed diet, and an energy-dense diet. Only the processed diet has been found to be associated with CRCs in the Malaysian population. The association between dietary patterns and fecal microbiota could be further investigated to address lifestyle modification for CRC prevention.

## Data Availability

All data generated or analysed during this study are included in this published article.

## Abbreviations

BMI: Body mass index
BMR: Basal metabolic rate
CL: Confidence limit
CRC: Colerectal cancer
DEXA: Dual-energy X-ray absorptiometry method
DFSA: High-dairy, high-fruit and vegetable, high-starch, low-alcohol diets
EFA: Exploratory factor analysis
EI: Energy intake
CRC: Colorectal cancer
FFQ: Food frequency questionnaire
HC: Hip circumference
HCA: Heterocyclic amines
HCTM UKM: Hospital Canselor Tuanku Muhriz Universiti Kebangsaan Malaysia
HUSM: Hospital Universiti Sains Malaysia
MyFCD: Malaysian Food Composition Database
NHMS: National and Health Morbidity Survey
NOC: N-nitroso-compound
OR: Odds ratio
PAH: Polycyclic aromatic hydrocarbons
PAL: Physical activity level
PS: Power and sample size
RR: Relative risk
US: United States
WC: Waist circumference
WHO: World Health Organization
